# Fractional flow reserve: a clinical perspective

**DOI:** 10.1007/s10554-017-1159-2

**Published:** 2017-06-02

**Authors:** David Corcoran, Barry Hennigan, Colin Berry

**Affiliations:** 1 West of Scotland Heart and Lung Centre, Golden Jubilee National Hospital, Clydebank, UK; 2 BHF Glasgow Cardiovascular Research Centre, Institute of Cardiovascular and Medical Sciences, University of Glasgow, 126 University Place, Glasgow, G12 8TA UK

**Keywords:** Fractional flow reserve, Coronary physiology, Stable angina, Myocardial infarction, Optimal medical therapy, Coronary revascularisation

## Abstract

Fractional flow reserve (FFR) is a reference invasive diagnostic test to assess the physiological significance of an epicardial coronary artery stenosis. FFR-guided percutaneous coronary intervention in stable coronary artery disease has been assessed in three seminal clinical trials and the indications for FFR assessment are expanding into other clinical scenarios. In this article we review the theoretical, experimental and clinical basis for FFR measurement. We place FFR measurement in the context of the comprehensive invasive assessment of coronary physiology in patients presenting with known or suspected angina pectoris in daily clinical practice, and review the recent developments in FFR assessment.

## Introduction

Coronary artery disease (CAD) is a significant cause of morbidity and mortality worldwide [1]. Traditionally, angina pectoris is attributed to obstructive epicardial coronary atherosclerotic plaque, which results in myocardial ischaemia due to supply-demand mismatch. However, up to 40% of patients presenting with angina have no visual evidence of obstructive epicardial CAD on invasive coronary angiography (ICA) [2]. The increasing use of invasive diagnostic tests to assess key parameters of coronary physiology at the time of ICA, namely coronary pressure, flow and microvascular resistance assessment are providing new, clinically-relevant diagnostic information [3]. Additionally, coronary endothelial dysfunction may result in angina, and this may be assessed with vasoreactivity testing [4]. Grüntzig first recognised the importance of assessing the physiological significance of coronary lesions, measuring the resting trans-lesional gradient pre- and post-balloon angioplasty [5]. However, the bulky balloon catheter lead to an overestimation of trans-lesional gradients, resting measurements were used due to no available hyperaemic agent, and the absolute trans-lesional gradient was measured rather than the relative reduction in perfusion pressure. With technical advances, these barriers have almost completely been overcome [6–8].

**Fig. 1:**
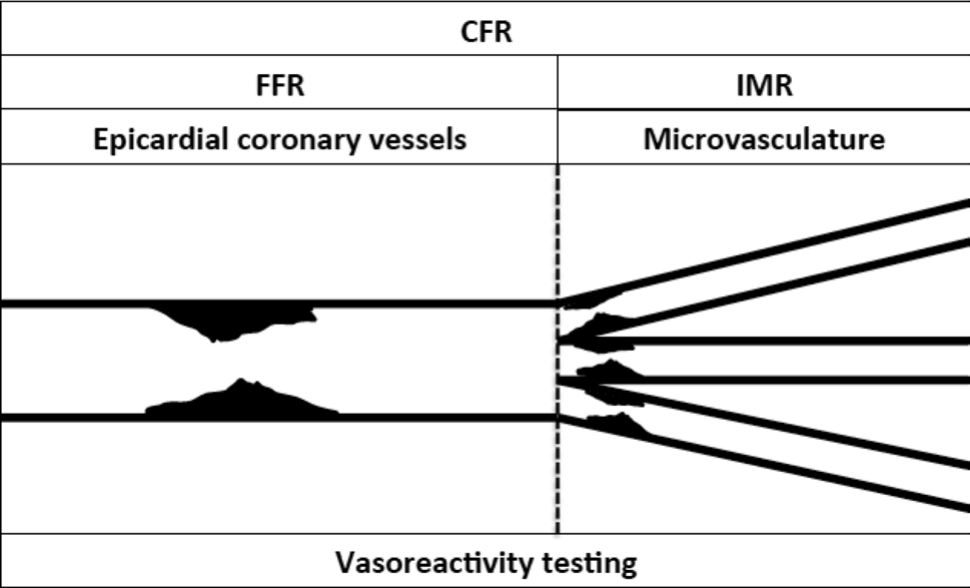
Schematic representation of the comprehensive assessment of the structural and functional components of the epicardial and microvascular compartments of the coronary tree. CFR represents the vasodilatory capacity of the epicardial vessel being interrogated and the microvasculature that it subtends. FFR is the reference standard for the assessment of the functional significance and an epicardial coronary stenosis. The IMR is a specific measure or the microvascular resistance being interrogated. Endothelial function, and epicardial and microvascular spasm may be tested for with vasoreactivity testing. *FFR* fractional flow reserve, *IMR* index of microcirculatory resistance, *CFR* coronary flow reserve

The coronary vasculature may be artificially divided into three compartments [9]. The epicardial coronary arteries (diameter >500 µm) are predominantly capacitance vessels and in the healthy state offer little resistance to blood flow. The coronary microvasculature is the predominant site of auto-regulation of myocardial blood flow and resistance to coronary blood flow. Pre-arterioles (diameter 100–500 µm) are comprised of proximal and distal vessels, which are most responsive to changes in flow and pressure respectively. Pre-arterioles regulate perfusion pressure into the subtended arteriolar compartment. Arterioles (diameter <100 µm) match myocardial blood supply and demand and are the predominant site of metabolic regulation of myocardial blood flow [10, 11]. It is increasingly appreciated that structural abnormalities limiting myocardial blood flow may occur not just in the epicardial vessels but also in the microvasculature, and that functional abnormalities may also result in angina [12]. Comprehensive assessment of the structural and functional components of each coronary compartment may be indicated, especially in the clinical scenario of ‘angina with normal coronaries’. Fractional flow reserve (FFR) is the reference-standard method to define flow-limiting lesions in the epicardial coronary compartment. FFR use is increasing [13, 14], and the European Society of Cardiology gives FFR a class 1A indication for the assessment of intermediate severity stenosis (defined as 50–90% diameter stenosis) [1, 15]. In the USA, the increasing frequency of FFR guidance has also been stimulated by recent appropriate use criteria and some private insurance companies require evidence of ischaemia by FFR assessment if percutaneous coronary intervention (PCI) is to be undertaken [16] (Fig. 1).

## Assessment of epicardial coronary artery stenosis and angiographic-physiological discordance

ICA is traditionally the standard reference test for the assessment of obstructive epicardial CAD. However, there is a poor correlation between visually-assessed anatomical stenosis severity on ICA and the physiological significance of a stenosis in terms of reduction in myocardial blood flow [17]. In study of 1000 patients who underwent ICA, intra-vascular ultrasound (IVUS), and FFR, it was determined that quantitative coronary angiography (QCA) had a diagnostic accuracy of 66% compared with FFR. A significant QCA diameter stenosis (>50%) and a non-significant FFR (>0.80) was predicted by older age, non-left anterior descending artery lesions, absence of plaque rupture, shorter lesion length, larger minimum luminal cross-sectional area (MLA), less plaque burden, and worse angiographic minimal lumen diameter. The predictors for a non-significant QCA diameter stenosis (<50%) with a significant FFR < 0.80 were younger age, LAD lesions, presence of plaque rupture, smaller MLA, and larger plaque burden [18].

## Fractional flow reserve: theory, experimental validation and practical considerations

FFR is a pressure-derived index of the maximal blood flow in an epicardial coronary vessel in the presence of a stenosis compared to the maximum flow in the hypothetical absence of a stenosis. At maximal hyperaemia during coronary vasodilator administration, when coronary resistance is minimised, blood flow is approximately linearly related to coronary pressure within the physiological range of coronary perfusion pressures [19].

**Fig. 2:**
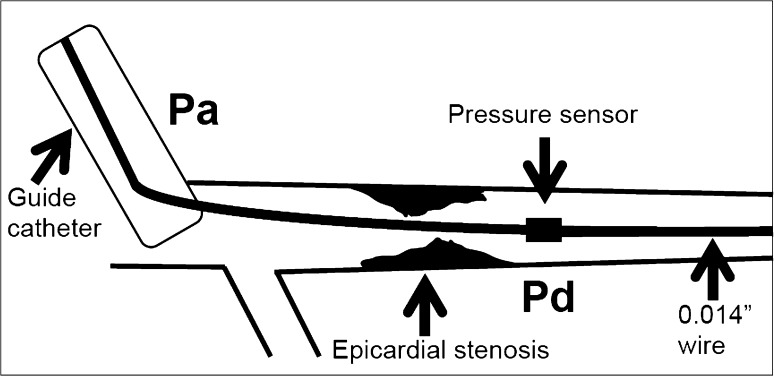
Schematic representation of fractional flow reserve measurement. *Pa* proximal (aortic) pressure, *Pd* distal coronary pressure

In order to determine the physiological significance of a coronary lesion, a pressure-sensitive 0.014″ coronary wire is positioned distal to an epicardial coronary lesion. The pressure distal to the coronary stenosis is obtained from a pressure sensor 3 cm proximal to the tip of the wire. The pressure proximal to the stenosis is obtained from the coronary guide catheter, which sits at the ostium of the coronary artery proximal to any obstruction. A bolus of 200–300 µg of intra-coronary glyceryl trinitrate is administered to provoke epicardial vasodilation, and counteract any coronary wire-related spasm which may mimic a stenosis. Maximal hyperaemia of the microvasculature is most commonly induced with intravenous (140 µg/kg/min for at least 2 min) or intra-coronary (100–200 µg) adenosine. If there is a left-dominant system or a right-dominant right coronary artery under study, then escalating doses may be used starting with 50 µg to avoid prolonged atrioventricular nodal block. FFR is calculated from the mean distal coronary pressure (Pd) indexed to the mean aortic pressure (Pa) obtained simultaneously at maximal hyperaemia: FFR = Pd/Pa [19]. The theoretical FFR value in a normal epicardial vessel without obstruction to blood flow is a ratio of 1.0. A clinical threshold of ≤0.8 is used to define a significant coronary stenosis [1] (Fig. 2).

In routine clinical practice, the myocardial FFR (FFRmyo) is measured, defined as the relative blood flow to the myocardium subtended by the coronary vessel being interrogated. By measuring the coronary wedge pressure (Pw) during maximal hyperaemia, the influence on myocardial blood flow from sources other than the epicardial artery (such as collateral and venous flow) can theoretically be accounted for. This is impractical in most cases as it requires balloon inflation to interrupt anterograde flow and so Pw measurement is predominantly performed in vessels undergoing PCI or in a research setting. The coronary FFR (or FFRcor) incorporates wedge pressure into its calculation, where FFRcor = [Pd − Pw]/[Pa − Pw]. In clinical practice, the measurement of Pw does not significantly alter the measured FFR, and does not influence the decision for revascularisation [20]. In its original derivation, the calculation of FFR accounted for right atrial pressure (Pv) where, FFRmyo = [Pd − Pv]/[Pa − Pv] [19]. Pragmatically, venous pressure is not usually measured in clinical practice to avoid central venous catheterisation, and Pv was not used for FFR calculation in the landmark clinical trials [21–23]. In usual practice, Pv has negligible influence on the measured FFR [24].

The experimental validity of determining relative blood flow (i.e. FFR) from pressure measurements obtained at hyperaemia was initially investigated in a canine model [25]. The first clinical validation in 45 patients, compared FFR against a gold standard of three non-invasive ischaemia tests, interrogating different aspects of the ischaemic cascade: electrical (exercise electrocardiogram testing), perfusion (myocardial perfusion thallium scintigraphy) and contractile (dobutamine stress echocardiography) [26]. In patients with a negative FFR (>0.75), 21 out of 24 patients had no evidence of inducible ischaemia on all three of the non-invasive modalities.

**Fig. 3:**
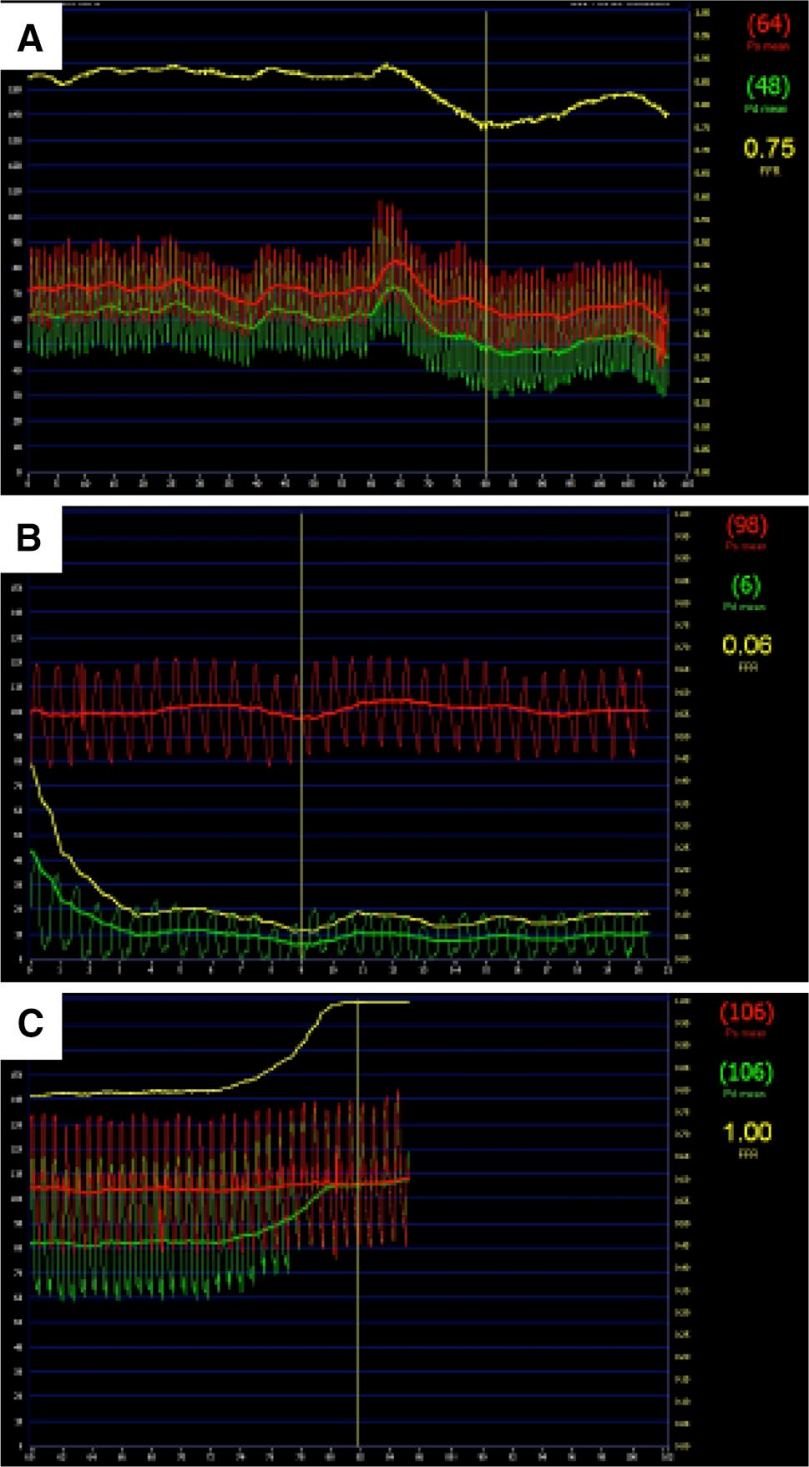
**a** FFR recording demonstrating the interrogation of a significant coronary lesion with an FFR value below the clinical ischaemic threshold of 0.8. **b** Measurement of a coronary wedge pressure. **c** Pull-back assessment for pressure wire ‘drift’ following FFR measurement

Practical considerations in the approach to FFR measurement are always important. The operator should carefully ensure calibration steps with the console. The angiographic catheter should be co-axial and not wedged. The FFR diagnostic wire should be positioned 6–9 cm from the guide catheter in the artery of interest (or in the distal half of the vessel). Resting pressure should be measured first before induction of hyperaemia, and if there are serial lesions, a pull-back recording should be made. Finally, the possibility of sensor-drift should be checked and if evident, calibration should be repeated (Fig. 3).

## Induction of maximal hyperaemia

Only during hyperaemia when the microvascular resistance is minimised does the coronary pressure–flow relationship become linear within the physiological range of blood pressure, which is a basic assumption for FFR. Hence, achieving maximal hyperaemia is key to avoiding false-negative FFR values. Adenosine is most commonly used in clinical practice, inducing vasodilation via agonism of the A_2A_ receptor, and thus minimising coronary resistance [27]. Intravenous infusion or intra-coronary bolus may be used [28]. A key advantage of intravenous infusion is that ‘pull-back’ assessment may be performed to ascertain the anatomical location of a ‘step-up’ in pressure gradient occurs (i.e. the location of a significant epicardial lesion) [29].

An appropriate response to adenosine should be confirmed at the time of administration (i.e. Increase in heart rate, reduction in systemic blood pressure and typical symptomatology), otherwise there is a risk of false-negative FFR values [30]. A common confounder is caffeine, a potent A_2A_ receptor antagonist [27], which reduces the vasodilatory response to adenosine and thus results in submaximal hyperaemia. Patients are routinely recommended to avoid caffeine consumption for >24 h prior to FFR assessment [31]. If there is a lack of response to adenosine, it may be useful to increase the dosage (i.e. 140, 175, and 210 μg/kg/min) to ensure maximal hyperaemia is achieved, and there is evidence that caffeine antagonism is overcome with high dose adenosine [32]. A number of vasodilator agents other than adenosine have been investigated, including intravenous regadenoson (a specific A_2A_ receptor agonist) [33], and intra-coronary sodium nitroprusside [34], nicorandil [35], nitrate [36], and papaverine [31]. These agents produced approximately similar FFR values compared to adenosine [35]. Radiographic contrast medium injection can also induce hyperaemia, and has been proposed as an alternative to adenosine hyperaemic FFR. The Continuum of Vasodilator Stress From Rest to Contrast Medium to Adenosine Hyperemia for Fractional Flow Reserve Assessment (CONTRAST) Study demonstrated an 85.8% diagnostic accuracy in reference to FFR at a cut-off of 0.83 whereas alternative resting pressure indices, including resting Pd/Pa and the instantaneous wave free ratio (iFR^®^), demonstrated a lower diagnostic accuracy of <80% in the 763 lesions studied [30]. This result supports a conclusion that diagnostic accuracy increases with increasing magnitude of hyperaemia.

## Resting pressure indices

**Fig. 4:**
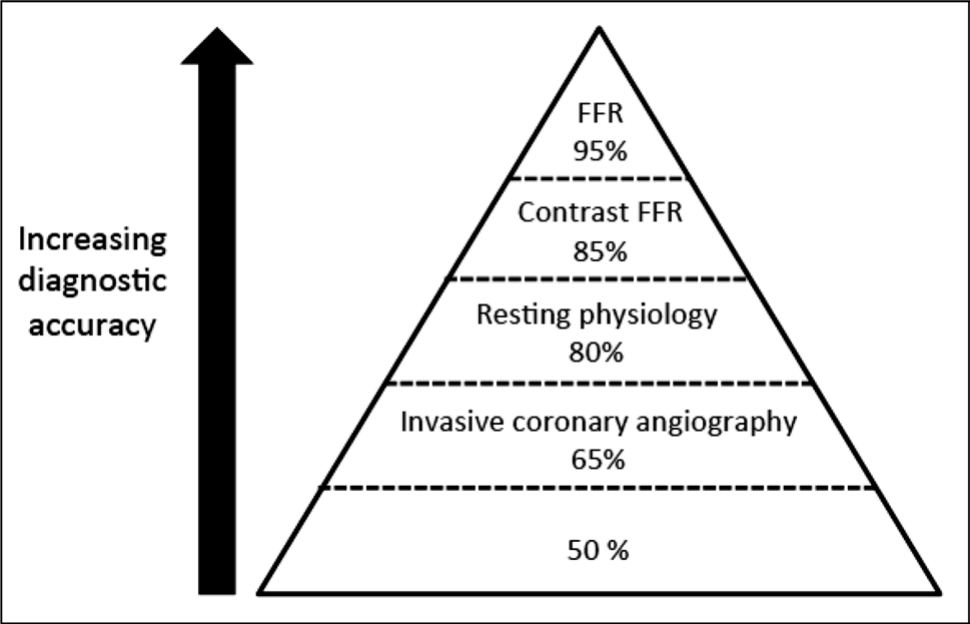
Pyramid of diagnostic accuracy with invasive assessments of coronary physiology. Adapted from Johnson et al. [63]

The interest in resting physiology to estimate the functional significance of an epicardial stenosis was first explored by Grüntzig [5], and has recently been revisited. Compared to the FFR reference metric, alternative resting indices achieve a diagnostic accuracy of approximately 80% (80.4 and 82.5% from the RESOLVE and ADVISE-II analyses respectively) when compared to FFR [37, 38]. This interest has been prompted by the desire to avoid adenosine hyperaemia due to short-lived patient side-effects including flushing and dyspnoea, and the cost and limited availability of adenosine in some parts of the world. ‘Whole-cycle resting Pd/Pa′ is the distal coronary pressure indexed to aortic pressure without the induction of hyperaemia, whereas iFR^®^ is defined as the pressure ratio at rest during a time interval starting 25% into diastole and ending 5 ms before onset of systole. iFR^®^ is calculated using proprietary software, whereas resting Pd/Pa is available generically using any FFR system. For an FFR ischaemic threshold of ≤0.75, whole-cycle resting Pd/Pa cut-offs of ≤0.85 to ≥0.93 have been proposed (with a positive predictive value of 95% and negative predictive value of 95.7% respectively) [39]. iFR^®^ was initially proposed by the ADVISE investigators as an adenosine-free test with a threshold of 0.83 being equivalent to the clinical FFR threshold of 0.80 [37, 40], however, research by the VERIFY investigators confirmed that iFR^®^ is lowered significantly when measured during intravenous adenosine infusion [41]. This threshold has since been revised to 0.89 with a diagnostic accuracy of around 82.5% [38]. Using a hybrid algorithm which incorporates hyperaemic FFR measurements, patients whose iFR^®^ falls in the 0.86–0.93 range receive adjunctive adenosine and undergo full FFR assessment due to diagnostic uncertainty in the so-called ‘adenosine zone’. This accounts for up to 35–45% of cases undergoing assessment but results in greater diagnostic accuracy versus FFR [42–44]. Clinical trials designed to assess health outcomes with iFR versus FFR-guided management are ongoing [45] (Fig. 4).

## Morphological coronary stenosis and patient features influencing fractional flow reserve

Morphological characteristics of an epicardial coronary stenosis are relevant to the reduction in myocardial blood flow that a given lesion may cause. The pressure drop across a stenosis is defined by: ΔP = fQ + sQ^2^, where ΔP = change in pressure, f = the viscous frictional forces along a lesion [increasing linearly with blood flow (Q) as explained by Poiseuille’s Law], and s = the separation forces due to eddy formation at the exit from a stenosis (increasing with the square of blood flow, as explained by Bernoulli’s Law) [46, 47]. Accordingly, lesion-specific factors may impact on the measured FFR: (1) lesion length has a strong inverse correlation with FFR value, with a length of >20 mm suggested as the strongest morphological determinant of functional significance [48, 49]; (2) increasing lesion diameter stenosis (assessed by QCA) correlates with lower FFR values [48]; (3) increasing lesion complexity (assessed by QCA) also predicts greater pressure losses due to flow separation and friction [50, 51]; (4) lipid-rich necrotic core coronary plaques have been associated with significant FFR values, independent of diameter stenosis [52]. A potential explanation is that these vulnerable plaques have reduced vasodilator capacity and are therefore more likely to be ischaemia-inducing (and therefore have significant FFR values). (5) Lesion location: as the volume of tissue that is subtended by a coronary stenosis increases so may the trans-lesional pressure gradient, and reduction in distal coronary pressure [53].

Patient-related variables may affect the influence the FFR value: (1) for any given angiographic stenosis severity, the measured FFR values are higher with increasing age [54]. A putative explanation is age-related cardiac changes such as interstitial fibrosis may result in coronary microvascular dysfunction; (2) for the same degree of angiographic stenosis severity, women are more likely to have higher FFR values [55, 56]. Potential explanations for this observation include an increased prevalence of microvascular dysfunction in females compared to males, and that females have a lower body surface area and lower myocardial mass subtended by each coronary artery compared to males; There is an inverse linear correlation between the measured FFR value and the mass of myocardium subtended by the vessel being interrogated [53].

Microvascular dysfunction is also relevant. If there is reversible microvascular dysfunction e.g. reflecting recovery of microvascular function within a culprit artery post-myocardial infarction, then Pd would expectedly reduce as reversible microvascular function improves. In other words, FFR may be inappropriately high. On the other hand, if microvascular dysfunction is fixed, then the FFR value would be expectedly stable. Therefore, the key question is whether or not the microcirculatory dysfunction is fixed or reversible. In this regard, measuring the index of microcirculatory resistance (IMR = mean P_d_ × mean transit time, during hyperaemia), and whether or not there is reversibility, as reflected by the resistance reserve ratio [RRR = basal resistance index (mean Pd × mean transit time, at rest)/IMR], may be helpful [57].

These lesion- and patient-specific variables are clinically relevant [58]: in a young patient, a proximal focal severe lesion in coronary vessel subtending a large myocardial mass, the losses due to separational forces will predominate. These lesions are more likely to have relatively preserved resting Pd/Pa values, with a significant increase in coronary flow across the lesion with hyperaemia resulting in a significant gradient and reduced FFR values. In contrast, in long moderate lesions, the frictional losses will predominate. There may be a relatively lower resting Pd/Pa and a more modest reduction in FFR with hyperaemia.

## Rational for the ischaemic threshold based on the DEFER and FAME trials

In the original validation study [26], the FFR threshold for discriminating clinically significant lesion-level ischaemia was found to be 0.75. This threshold was confirmed in The Fractional Flow Reserve to Determine the Appropriateness of Angioplasty in Moderate Coronary Stenosis (DEFER) trial [21]. 325 patients who were planned to undergo PCI underwent FFR measurement. Patients with an FFR ≥ 0.75 were randomised to deferral of PCI (defer group) or to undergo PCI (perform group), whereas those with an FFR < 0.75 underwent PCI as planned (reference group). There was no significant difference in the primary endpoint of absence of adverse cardiac events at 24 months follow-up in the defer versus perform groups, demonstrating that patients with negative-FFR values (defined as >0.75) did not benefit from PCI when compared to medical therapy. At 15-years follow-up, there remained no significant difference in the rate of death in patients with functionally insignificant lesions (FFR > 0.75) that were managed with medical therapy, and there was a lower rate of myocardial infarction (MI) in the defer group compared to the perform group (2.2% vs. 10.0%, p = 0.03) [59].

A meta-analysis of study-level (n = 9173) and patient-level (n = 6961) data similarly found the optimal FFR threshold for a composite endpoint of death, MI and revascularisation to be 0.75 [60]. As opposed to a binary cut-off, the measured FFR value has prognostic importance, with a spectrum of increasing clinical events with decreasing FFR value. This is one reason for why measuring resting pressure indices alone, or adopting a hybrid strategy, may be less informative overall, since FFR is not routinely measured with these approaches. In clinical practice, patients with FFR values close to the treatment threshold i.e. 0.81–0.85 have a higher likelihood of future major adverse cardiac events compared to patients with a near normal FFR value i.e. 0.96–1.0 [60]. Patients with a higher risk profile according to measured FFR may therefore warrant more aggressive secondary prevention strategies compared to patients with near normal values.

To reliably exclude the presence of functionally significant stenoses, a threshold of ≤0.80 is routinely used in clinical practice to increase measurement sensitivity. A threshold of ≤0.80 was used in the two FAME outcomes trials of FFR-guided PCI [22, 23]. This has produced diagnostic uncertainty for patients with FFR values of 0.75–0.80 inclusive. For patients in this ‘grey zone’, physician decision-making informed by all clinical information (e.g. anginal symptoms and non-invasive evidence of ischaemia) is especially important. A retrospective analysis included 1459 patients with proximal single-vessel disease and FFR values in the grey zone (defined as 0.76–0.80) and the neighbouring FFR strata (0.70–0.75, and 0.81–0.85) [61]. 449 (30%) patients underwent PCI, and 1010 (70%) were treated with medical therapy alone. In patients treated with medical therapy alone, there was a progressive decrease in MACE rates with increasing FFR value. In the grey zone, there was a trend towards an increased rate of death or MI in patients managed medically compared to PCI (25 vs. 9, p = 0.06). The Trial in Stable Intermediate Coronary Lesions and Grey-zone FFR Values (gzFFR) (ClinicalTrials.gov Identifier: NCT02425969) will further inform this issue. In this trial, 110 patients with stable anginal symptoms who have undergone ICA and been found to have an epicardial coronary stenosis with a ‘grey zone’ FFR (0.75–0.82 inclusive) will be enrolled. Participants will be randomly assigned (1:1) to either optimal medical therapy (OMT) or PCI. Patients will undergo comprehensive physiological assessment with acquisition of Doppler coronary flow and resistance data, as well as repeated FFR measurements with incremental doses of adenosine. Stress perfusion cardiac magnetic resonance imaging at 3.0 T will be used to define the prevalence of inducible perfusion abnormalities in the myocardium subtended by the study vessel. The primary outcome is angina severity at 3 and 12 months with a key secondary endpoint of major adverse coronary events (MACE) at 3 and 12 months.

Another key consideration is which lesions should undergo FFR interrogation. The ESC guidelines define an intermediate stenosis as 50–90% [1]. In a diagnostic study of 200 patients [62], 47% of lesions defined as >70% diameter stenosis were FFR-negative and 13% of lesions graded as <30% were FFR-positive. This suggests that rather than only using FFR for ‘intermediate’ stenoses, more discrete areas of coronary plaque should be interrogated, especially in younger patients, in epicardial vessels with proximal lesions subtending a large myocardial mass (namely left main stem and left anterior descending artery lesions), or lesions of long length.

## Fractional flow reserve reproducibility

FFR measurement has been shown to be highly reproducible in clinical practice despite differences in the route of administration of hyperaemic agent, and variation in the hyperaemic agent used. The VERification of Instantaneous wave-Free ratio and FFR for the assessment of coronary artery stenosis severity in everydaY practice (VERIFY) [41] was a prospective study of 206 patients with an indication for FFR measurement. FFR was measured using intravenous adenosine (140 µg/kg/min administered for 2 min), with repeat measurements made after a 2-min rest period. FFR data were assessed by a central laboratory, with FFR reproducibility high (r^2^ = 0.98) and narrow limits of agreement (−0.04 to −0.04). Test–retest reliability for FFR in the CONTRAST Study was 0.019 indicative of a high reproducibility [63].

A key assumption of FFR measurement is that of maximal hyperaemia. Pd/Pa value may fluctuate during a hyperaemic recording and the minimum FFR may not be the same as the steady-state FFR. A reanalysis of the VERIFY dataset demonstrated that despite fluctuating haemodynamics, the minimum measured FFR value is the most reproducible. The authors have developed a novel ‘smart minimum’ algorithm to select out the highest quality FFR data within a recording, which may help cardiologists identify the minimum FFR value for clinical decision-making [64].

## Fractional flow reserve-guided percutaneous coronary intervention in stable coronary artery disease: clinical outcomes studies

Following DEFER [21], the FFR versus Angiography for Guiding Percutaneous Coronary intervention (FAME) [22] and Fractional Flow Reserve-Guided PCI versus Medical Therapy in Stable Coronary Disease (FAME-2) [23] clinical trials have established FFR-guided PCI as the standard of care in patients undergoing invasive management.

FAME tested the hypothesis that PCI guided by FFR measurement in patients with stable angina and multivessel CAD (defined as >50% stenosis in ≥2 main epicardial vessels) would alter lesion classification and improve health and economic outcomes. After the decision to undertake PCI based on ICA, 1005 patients were randomised to FFR-guided PCI (PCI performed if FFR ≤ 0.8) or to continue with PCI guided by visual interpretation of the angiogram alone. The composite primary outcome of death, MI or repeat revascularisation at 1 year was lower in the FFR-guided group than in the angiography-guided group, [13.2% (67 patients) vs. 18.3% (91 patients), p = 0.02]. This difference was sustained at 2 years follow-up [65]. At 5 years follow-up, there was no difference in the primary endpoint between the FFR- and angiography-guided groups (28% vs. 31%, p = 0.3), but the absolute difference in events persisted, and this was driven by the difference in cardiac mortality [66].

FAME-2 enrolled 1220 patients with stable CAD who were undergoing invasive management and being considered for PCI of one or more angiographically severe stenosis. In the subset of patients with lesions with an FFR ≤ 0.80 that were amenable to PCI (n = 888), patients were randomised (1:1) to PCI of all lesions with OMT versus OMT alone without PCI. Patients with lesions with an FFR > 0.80 were not randomised but included in a follow-up registry involving OMT alone. On the recommendation of the Data and Safety Monitoring Committee, the trial was stopped prematurely due to a statistically significant reduction in hospital re-admission for urgent coronary revascularisation in the OMT group. It has been proposed that urgent revascularisation is a ‘soft’ endpoint, and as patients were aware they had a coronary lesion which had not undergone PCI, this may have influenced the likelihood of re-presentation to hospital [67]. Urgent coronary revascularisation for the primary outcome was defined as urgent unplanned hospital admission with persistent or increasing symptoms with or without ECG evidence of ischaemia (26.8%) or elevated cardiac biomarker levels (21.4%), and that the revascularisation be performed within 24 h of admission. Cardiologists blinded to the treatment group assignment adjudicated this outcome. After follow-up to 2 years, the observed between-group difference in the primary outcome was maintained [68].

## Fractional flow reserve in the diagnostic and treatment decision-making pathway for stable coronary artery disease

FAME and FAME-2 provide a robust evidence base for the use of FFR-guided PCI in patients undergoing invasive management. FFR is also impactful earlier in the diagnostic pathway. The diagnostic pathway for patients presenting with chest pain is complex, with variations in practice depending on the physician certainty of a clinical diagnosis of angina pectoris, and limitations of non-invasive ischaemia testing due to local availability and diagnostic accuracy. The Does Routine Pressure Wire Assessment Influence Management Strategy at Coronary Angiography for Diagnosis of Chest Pain (RIPCORD) study was designed to assess whether routine FFR measurement during diagnostic ICA would impact the management of patients when compared with visual assessment of the angiogram alone [62]. 200 patients with stable angina who had been referred for ICA were enrolled in 10 UK centres. The ICA was visually interpreted and a management plan formulated by the treating cardiologist. FFR was then measured in any epicardial vessel ≥2.25 mm with a ≥30% stenosis. The management plan (‘OMT alone’, ‘PCI’, ‘coronary artery bypass grafting (CABG)’, or ‘more information required’) changed in 26% and the number and localisation of functionally significant stenoses was altered in 32%. Similarly, The Registre Français de la FFR (R3F) [69] enrolled 1075 consecutive patients from 20 centres who had stable angina who were undergoing ICA, and whom were found to have at least one intermediate coronary stenosis (defined as 30–65% stenosis). The results were consistent with those of RIPCORD, with the frequent reclassification of management with FFR-guided management compared to visual interpretation of the angiogram alone (47% of the cases). This evidence suggests that routine use of FFR at the diagnostic stage may improve the treatment decision-making for patients by correctly identifying functionally significant coronary lesions facilitating lesion-level tailored management. Whether this will result in improved clinical outcomes will be answered by a number of ongoing studies: The proposed RIPCORD 2 study [70] will randomise 1100 patients presenting with chest pain who are scheduled to undergo ICA to angiography-guided management or FFR-guided management; The Functional Testing Underlying Coronary Revascularisation (FUTURE) trial (NCT01881555) will randomise 1728 patients chest pain with multi-vessel coronary artery disease diagnosed on ICA, to angiography alone versus angiography and FFR-guided care, with a composite primary end point of death, MI, coronary revascularisation, and stroke at 1 year.

**Fig. 5:**
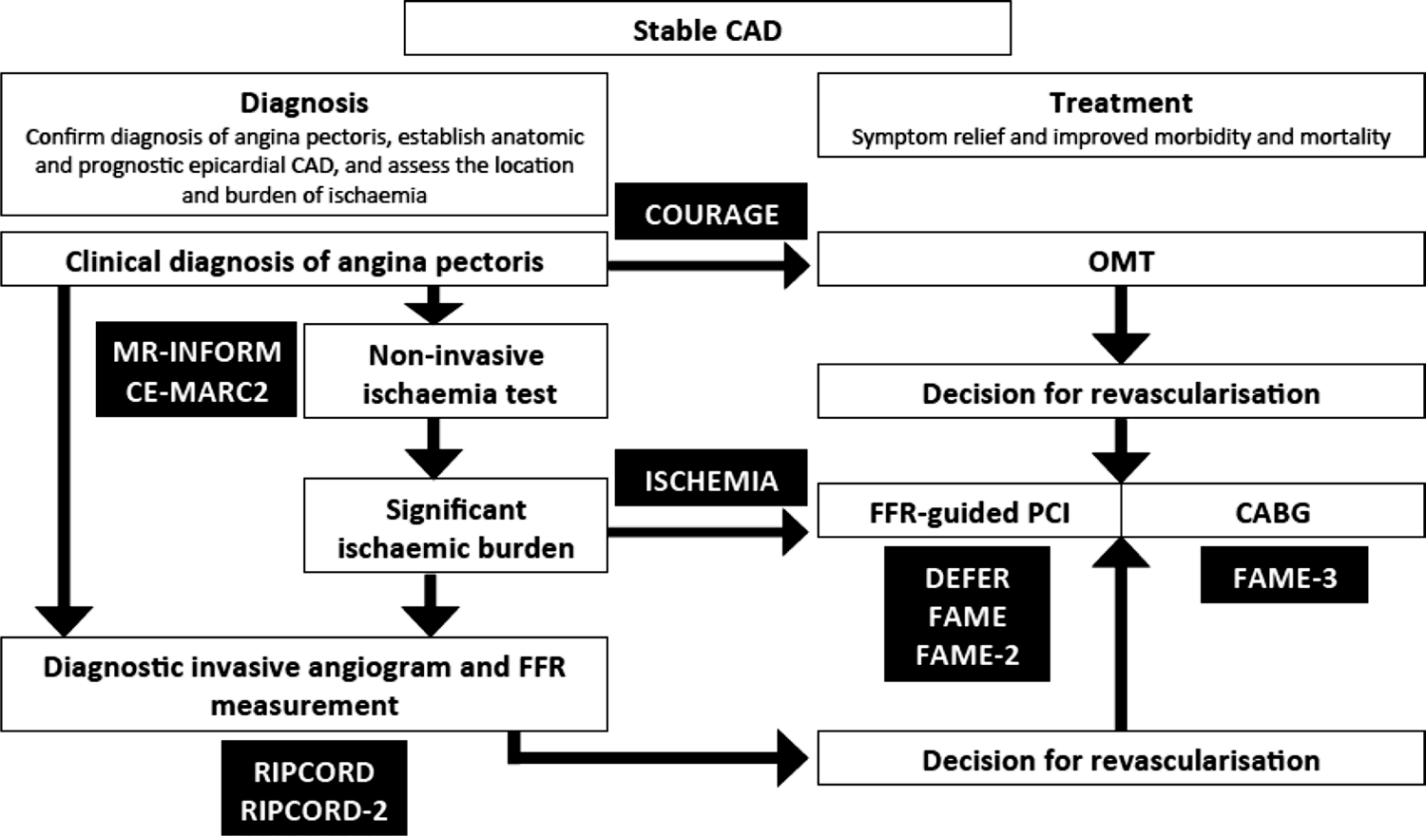
Indications for the use on FFR-guided care in the diagnostic and invasive management of patients with stable CAD, and the relevant completed and ongoing FFR-guided clinical trials (*black boxes*), MR-INFORM [102], CE-MARC2 [103], RIPCORD [62], RIPCORD-2 [70], COURAGE [71], ISCHEMIA, DEFER [21], FAME [22], FAME-2 [23], FAME-3

The RIPCORD study [62] confirmed that the management of patients with stable angina with visual-interpretation of the ICA alone is flawed. Two seminal studies, The Optimal Medical Therapy with or without PCI for Stable Coronary Disease (COURAGE) trial [71] and The Synergy between PCI with Taxus and cardiac surgery (SYNTAX) study [72] trials, investigated revascularisation of patients with stable CAD. Both studies used visual-interpretation of angiography alone to define significant epicardial coronary stenosis. This will have almost certainly led to incorrect classification of functionally significant flow-limiting lesions. The results of these studies may have produced different results had FFR-guided management been performed:

1. The COURAGE trial [71] randomised 2287 patients with stable angina and visually-assessed severe coronary stenosis to PCI or no PCI. At a median follow-up of 4.6 years, there was no difference in the composite primary endpoint of death and MI. A key sub-study of COURAGE, demonstrated that patients with evidence of inducible ischaemia on SPECT imaging (≥10% ischaemic myocardium) (i.e. Those patients whom FFR-guided assessment would classify) who were randomised to PCI, had significant reductions in inducible ischaemia and in the annual rate of death and MI [73]. This forms the basis for the ‘ischaemia hypothesis’ and that PCI may improve outcomes in patients with stable CAD and a moderate to large ischaemic burden. This question will be investigated by The International Study of Comparative Health Effectiveness with Medical and Invasive Approaches (ISCHEMIA) trial (NCT01471522). ISCHEMIA (n = 8000) tests the hypothesis that an initial invasive strategy of coronary angiography followed by PCI if feasible, in addition to OMT, will reduce the primary composite endpoint of cardiovascular death or non-fatal MI in patients with at least moderate ischaemia on stress imaging, compared with an initial conservative strategy of OMT alone with coronary angiography reserved for failure of OMT. The invasive strategy involves FFR-guided revascularisation of ‘intermediate’ coronary stenoses (50–80% diameter stenosis), routine revascularisation for lesions >80% severity and no revascularisation for lesions <50% severity. The trial is currently enrolling patients worldwide.
2. The SYNTAX study, randomised 1800 patients with left main stem and multivessel CAD to PCI or CABG [72]. SYNTAX demonstrated significantly increased rates of major adverse cardiac or cerebrovascular events (MACCE) at 12 months in those who underwent angiography-guided PCI compared to CABG (17.8% vs. 12.4%), failing to meet the criterion of non-inferiority for PCI. At 5-year follow-up in patients with a low SYNTAX score (an angiographically-defined anatomical scoring tool used to describe the complexity of epicardial atherosclerotic CAD) there was no difference in MACCE in the PCI and CABG groups (32·1% vs. 28·6%, p = 0·43). However, in patients with intermediate or high SYNTAX scores, there was significantly increased MACCE with PCI compared to CABG (intermediate score, 36·0% vs. 25·8%, p = 0·008; high score, 44·0% vs. 26·8%, p < 0·0001) [74]. Would the results of SYNTAX have been different had patients undergone FFR-guided PCI? The functional SYNTAX score only includes and scores lesions which are flow-limiting (FFR ≤ 0.80). Using this score, in 497 patients from the FAME cohort, 32% of patients were reclassified and moved to lower risk SYNTAX score tertiles [75]. A retrospective analysis of 627 patients undergoing FFR-guided CABG found FFR-guided CABG to be associated with fewer graft anastomoses, and did not result in a higher event rate (median 33.1 months follow-up). Further data will be provided by The Comparison of Fractional Flow Reserve-Guided Percutaneous Coronary Intervention and Coronary Artery Bypass Graft Surgery in Patients With Multivessel Coronary Artery Disease (FAME 3) study (NCT02100722) [76] which will randomise 1500 patients with multivessel CAD to FFR-guided PCI or CABG in patients with the primary endpoint of MACCE at 1-year follow-up (Fig. 5).

## Fractional flow reserve measurement in specific situations

The evidence for FFR-guided PCI in patients with stable CAD is supported by landmark clinical trials. In comparison, there is a smaller but growing literature on the role of FFR in specific situations encountered in daily clinical practice.

1. *Left main stem stenosis* FFR measurement of intermediate left main stem stenosis is associated with favourable outcomes. In 213 patients with angiographically equivalent left main stem stenosis, the prognosis of patients who were medically managed if the FFR > 0.80 was similar to those with FFR ≤ 0.80 who underwent CABG (5-year event-free survival 74.2% vs. 82.8% respectively, p = 0.50) [77]. A pooled meta-analysis of 6 studies (n = 525) investigating FFR-guided revascularisation of left main stem stenosis found no statistically significant difference between patients undergoing revascularisation if the FFR was significant, compared to patients being deferred intervention if the FFR was negative [78].
2. *Bifurcation lesions* With provisional stenting strategies, FFR assessment of the jailed side branch is feasible and safe: 110 patients undergoing PCI to bifurcation lesions had FFR measured in the jailed side branch. PCI to the side branch was performed if FFR < 0.75 [79]. FFR measurement was then repeated post-PCI and at 6 months follow-up, with post-PCI FFR ≥ 0.75 achieved in 92%. There was no significant change in the FFR values in the side branch lesions that underwent PCI compared to those that did not, and there was no difference in 9-month cardiac event rate (4.6 vs. 3.7% respectively, p = 0.7) when clinical outcomes of the study cohort were compared with 110 patients with similar bifurcation lesions undergoing angiographic-guided PCI.
3. *Surgical bypass grafts* FFR-guided PCI of intermediate stenosis in arterial and venous surgical bypass grafts is valid: a retrospective analysis included 223 patients with a history of previous CABG who presented with stable or unstable anginal symptoms, and who had evidence of an intermediate lesion in either an arterial or venous graft [80]. 65 patients underwent FFR assessment with PCI performed if the FFR ≤ 0.80, whereas 158 patients underwent angiography-guided PCI. At a median follow-up of 3.8 years, the primary outcome of MACCE was significantly lower in the FFR-guided compared angiography-guided group (18 vs. 77, p = 0.043).

## Fractional flow reserve measurement post-percutaneous coronary intervention

Rather than using visual interpretation of the angiogram alone to determine successful PCI, FFR may be measured post-PCI to inform this decision. A registry of 750 patients found a post-PCI value of <0.90 to predict worse outcomes [81]. As well as relating to the target lesion and inadequate stent deployment, a persistent gradient post-PCI may relate to diffuse atherosclerotic disease. This may be elicited by an FFR pullback recording along the length of the epicardial artery. Importantly, FFR pull-back recordings were not performed in this registry.

Following post-dilation, if there is a persistent gradient at the site of the target lesion, intra-coronary imaging with IVUS or optical coherence tomography (OCT) may be indicated [15]. Knowledge of the mechanism underlying a persistent gradient post-PCI is important to ascertain, and may inform further therapeutic decisions, as these patients may represent with ongoing angina symptoms. A multicentre study of 240 patients presenting with NSTEMI, randomised patients (1:1) to either angiographic- or OCT-guided PCI [82]. There was a statistically significant increase in the primary endpoint of the post-PCI FFR value in the OCT-guided compared to the angiographic-guided group (0.94 vs. 0.92, p = 0.005), with OCT revealing the persistent gradients to be related to stent under-expansion (42%), stent malapposition (32%), and incomplete lesion coverage (20%).

## Fractional flow reserve measurement in acute coronary syndromes

The diagnostic validity of FFR has been questioned in acute coronary syndrome (ACS) patients and guidelines state that FFR may be measured in intermediate coronary lesions >5 days after the index event [83]. This is due to the potential for athero-thrombotic milieu to result in culprit artery microvascular obstruction, and a reduced pharmacological vasodilator response (resulting in inadequate hyperaemia and thus a key assumption of FFR is invalid), and therefore ‘false negative’ FFR values.

The FAMOUS-NSTEMI trial [84] randomised 350 medically stabilised non-ST-elevation MI patients to either routine FFR-guided management versus standard invasive management. An initial treatment decision was made following ICA and before FFR measurement. Where feasible, FFR was then measured in all vessels with >30% diameter stenosis, but in patients randomised to angiographic guidance alone, the FFR results remained blinded. The primary outcome was the between-group difference in the proportion of patients allocated to optimal medical therapy alone, and this occurred more frequently in the FFR-guided group compared to the angiography-guided group (22.7% vs. 13.2%; p = 0.022). In other words, the use of FFR reduced revascularisation. There was no difference in MACE between the groups. As in the stable CAD population, a marked discordance was seen between the angiographic visual stenosis severity and functional significance defined by FFR. An FFR result was obtained in all participants with only two coronary dissections occurring due to the pressure wire, indicating routine FFR measurement was feasible and safe. There were no adverse events relating to intravenous adenosine.

FFR is not diagnostically valid when measured in the culprit artery of patients with an acute ST-elevation MI (STEMI). On the other hand, it may be diagnostically useful for the assessment of coronary disease in non-culprit arteries. Non-culprit FFR measurement (performed following treatment of the culprit lesion) in STEMI is reproducible when repeated at an interval of 35 ± 4 days [85]. Recent data on complete revascularisation of non-culprit vessels in the setting of STEMI has led to renewed interest in non-culprit FFR measurement in acute STEMI. The DANAMI-3-PRIMULTI trial [86] enrolled 627 patients presenting with STEMI and >1 angiographically significant stenosis in addition to the infarct-related artery. Patients were randomised 1:1 to infarct-related artery only PCI or to complete FFR-guided revascularisation prior to discharge. At a median follow-up of 27 months, there was a significantly increased primary endpoint (all-cause mortality, non-fatal re-infarction, and ischaemia-driven revascularisation of lesions in non-infarct-related arteries) in the infarct-related artery only PCI group compared to the complete FFR-guided revascularisation group (22% vs. 13%, p = 0.004). This functional assessment strategy is in contrast to the preventive PCI strategy of all non-culprit vessel lesions >50% visual diameter stenosis in the PRAMI trial [87]. The ongoing COMPARE-ACUTE (NCT01399736), COMPLETE (NCT01740479), FRAME-STEMI (NCT02715518), FULL REVASC (NCT02862119) and FAIO (NCT02637440) trials will provide further evidence on FFR-guided care in STEMI patients.

## Comprehensive invasive assessment of coronary physiology

FFR assesses the functional significance of an epicardial stenosis. Angina may result from abnormalities in other compartments, and the same coronary wire used to measure FFR, may be simultaneously used to interrogate the microvasculature (by indicator-thermodilution) allowing a more complete assessment of a patient’s coronary physiology at the time of ICA, while providing prognostic data to guide management [88]. In addition to IMR and RRR, the assessment of coronary flow reserve (CFR) provide complementary information and can aid in the differentiation of a patient’s symptoms due to focal or diffuse epicardial disease, microvascular disease, or both [89]. Although the importance of reduced CFR (secondary to epicardial or microvascular disease) in defining adverse prognosis has been clearly demonstrated [90], the role and prognostic importance of the interrogation of the microvascular compartment alongside the epicardial vessel with FFR measurement is yet to be defined.

## Non-invasive estimates of fractional flow reserve: what’s coming next?

Quantitative flow ratio (QFR) is a novel method for assessing the functional significance of intermediate coronary stenosis without the use of a coronary guidewire [91]. Following demarcation of vascular contours on orthogonal hyperaemic angiographic cine video-fluoroscopy acquisitions, a 3-dimensional QCA coronary reconstruction is formed using QFR software. A QFR value is then produced using computational fluid dynamics, corrected for the (user-defined) TIMI (Thrombolysis in Myocardial Infarction) frame count. In a validation cohort of 84 vessels from 73 patients with intermediate coronary lesions, QFR had a diagnostic accuracy for identifying lesions with an invasive FFR ≤ 0.8 of 87% [92]. The Wire-free Invasive Functional Imaging (WIFI) (NCT02795585) and the FAVOR II Europe (Holm NR, personal communication) studies will evaluate the feasibility and diagnostic accuracy of QFR measured using QCA during coronary angiography using virtual on-line reconstruction compared with invasively measured FFR as the reference.

Virtual FFR (vFFR) provides an estimated FFR value from rotational angiographic video-fluoroscopy. Hyperaemic acquisitions are not required. In a validation cohort of 19 patients vFFR and invasive FFR were measured. There was a good agreement between vFFR and the invasively measured FFR, with a deviation from the measured values of ±0.06 [93].

FFR may be estimated non-invasively from computed tomography coronary angiography (FFR-CT) using proprietary software which applies 3-dimensional blood flow simulations using the principles of computational fluid dynamics. The DISCOVER-FLOW study [94] estimated FFR-CT from 159 arteries in 103 patients who also underwent ICA and FFR measurement, and reported a diagnostic accuracy of 84.3%. The HEARTFLOW-NXT study [95] of 251 patients and 484 vessels similarly compared FFR-CT with invasively measured FFR and found a diagnostic accuracy of 86% on a per-vessel analysis. FFR-CT estimation may be limited by significantly calcific CAD, and technical factors which may reduce image quality (motion artifact, tachycardia, arrhythmia). The PLATFORM trial suggested that use of FFR-CT could lead to substantial reductions in unnecessary ICA and related health economic benefits [96, 97]. Exciting new studies, including ADVANCE and SYNTAX III, will probe the expanding clinical potential and utility of this technology [98, 99].

## Conclusion

For the functional assessment of an epicardial stenosis, a continuum exists from coronary angiography alone, to resting indices, to contrast-induced hyperaemia, to adenosine hyperaemic FFR. FFR-guided PCI in patients with stable CAD is well established with a robust evidence-base while the prognostic evidence for alternate indices is less well-established. FFR-guided PCI improves health outcomes and does so with improved cost-effectiveness compared to optimal medical therapy alone [100, 101]. Ongoing clinical trials will help to define the role of FFR assessment alongside non-invasive ischaemia testing in the diagnostic pathway of patients presenting with chest pain.
